# Virtual reality device training for extracorporeal membrane oxygenation

**DOI:** 10.1186/s13054-020-03095-y

**Published:** 2020-07-02

**Authors:** Georg Wolff, Raphael R. Bruno, Martina Reiter, Boris Kantzow, Malte Kelm, Christian Jung

**Affiliations:** 1 Division of Cardiology, Pulmonology and Vascular Medicine, Department of Internal Medicine, Medical Faculty, Heinrich-Heine University, Moorenstr. 5, 40225 Düsseldorf, Germany; 2 Getinge Group, Maquet GmbH, Kehlerstr. 31, 76437 Rastatt, Germany; 3 Weltenmacher GmbH, Binterimstraße 8, 40223 Düsseldorf, Germany

**Keywords:** Virtual reality, ECMO, Cardiohelp, VR, Priming

Extracorporeal membrane oxygenation (ECMO) is a last resort therapy for patients with terminal respiratory failure. In the current worldwide surge of critically ill patients with novel coronavirus disease (COVID-19), ECMO demand for the sickest of them is unprecedentedly high and management is very complex [1]. Highly trained healthcare personnel is essential to safely prime, implant, and operate ECMO devices [2]. Acquisition of such complex skillsets has always been difficult—especially for smaller hospitals with lower ECMO case counts [3]. During the pandemic, traditional face-to-face instructor-led training is additionally complicated by social distancing measures. Alternative and complementary ways of delivering high-quality training are thus desirable to increase personnel resources for ECMO services.

Virtual reality (VR) simulators are emerging as next-generation options in digital health to complement traditional training: VR training is largely independent of resources, location, and person-to-person contact; it integrates both teaching theory and practical application and allows unlimited repetition. Our research collaboration currently develops a prototype for VR training on an ECMO device (Fig. 1a): using a VR headset with controllers (Fig. 1b), trainees are immersed in a digital VR environment with a Getinge Cardiohelp® ECMO device (Fig. 1c+d). The virtual device is responsive to manual user input by movement of the body, head, and hands in the virtual space. A digital coach leads the trainee through a multi-layered didactic digital teaching program: beginners go through step-by-step video instructions and manually imitate each step on the ECMO device (Video 1); experts must perform tasks without any support (Video 2). Training includes sessions of the priming procedure of the device for use (Fig. 1c and Video 1) and configuring its program options (Fig. 1d and Video 2), each a complex sequence of single steps requiring specialized knowledge and manual skillsets. This VR prototype is ready to be evaluated for the ECMO priming procedure. It may be expanded to further content in the future, e.g., device troubleshooting or implantation. We are looking forward to reporting results of this innovative technology soon.

**Fig. 1:**
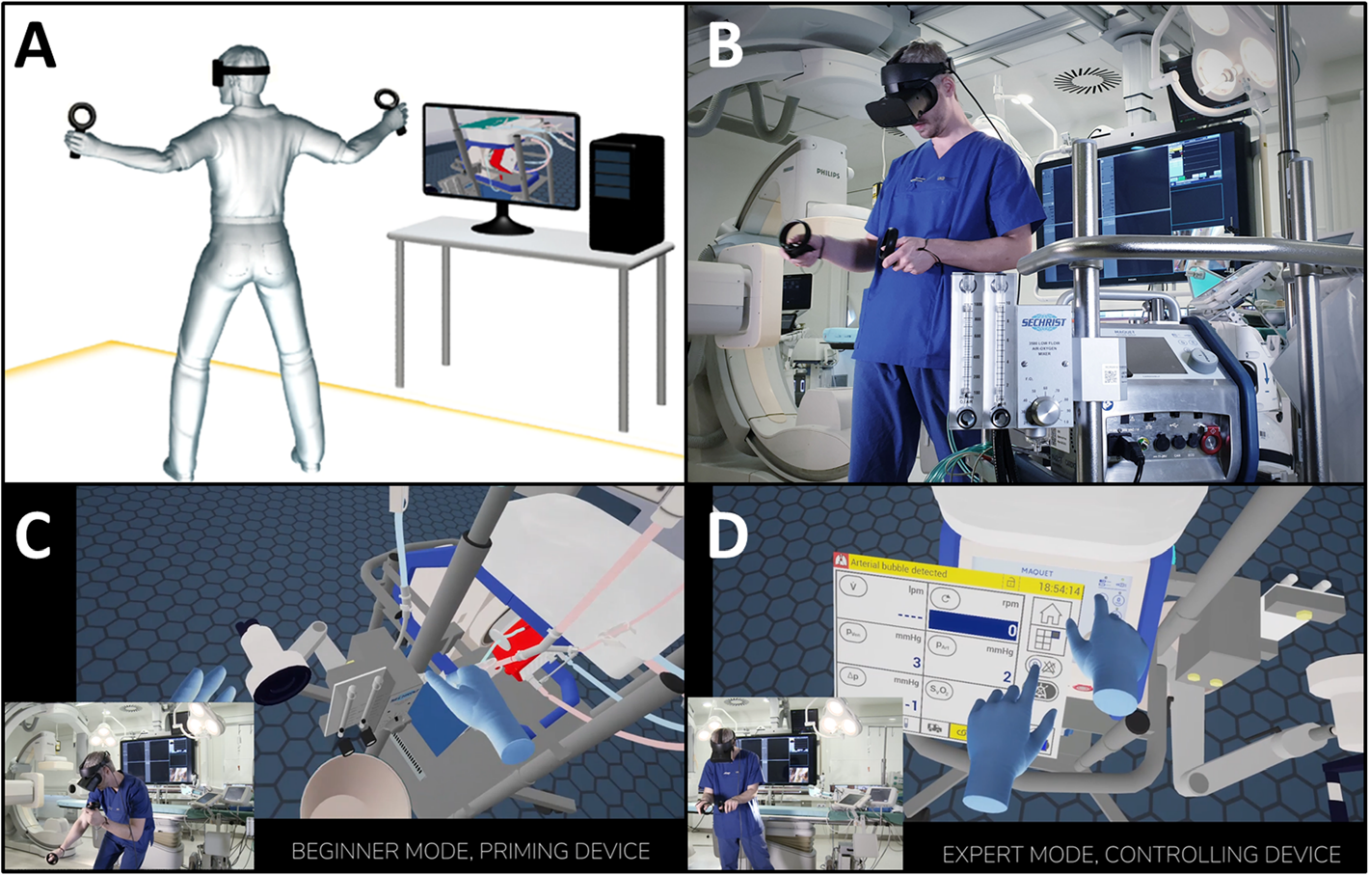
Virtual reality setup and Getinge Cardiohelp® ECMO training environment. Schematic drawing (**a**) and real-world shot (**b**) of a virtual reality setup, with scenes from the VR environment of priming (**c**) and controlling (**d**) the ECMO device

Virtual reality device training for extracorporeal membrane oxygenation promises to be a very valuable tool for health care personnel training—both during the pandemic and beyond.

## Supplementary information

**Additional file 1: Video 1.** Priming the device, beginner mode: Step-by-step instruction and manual repetition in Virtual Reality.

**Additional file 2: Video 2.** Controlling the device, expert mode: Configuring device options in Virtual Reality.

## Data Availability

All relevant data have been submitted, and additional graphic and video material is available upon request.
